# Brain Responses to Surprising Stimulus Offsets: Phenomenology and Functional Significance

**DOI:** 10.1093/cercor/bhab352

**Published:** 2021-10-20

**Authors:** R Somervail, R J Bufacchi, C Salvatori, L Neary-Zajiczek, Y Guo, G Novembre, G D Iannetti

**Affiliations:** Neuroscience and Behaviour Laboratory, Istituto Italiano di Tecnologia, 00161, Rome, Italy; Department of Neuroscience, Physiology and Pharmacology, University College London (UCL), WC1E 6BT, London, UK; Neuroscience and Behaviour Laboratory, Istituto Italiano di Tecnologia, 00161, Rome, Italy; Neuroscience and Behaviour Laboratory, Istituto Italiano di Tecnologia, 00161, Rome, Italy; Department of Computer Science, University College London (UCL), WC1E 6BT, London, UK; Neuroscience and Behaviour Laboratory, Istituto Italiano di Tecnologia, 00161, Rome, Italy; Neuroscience and Behaviour Laboratory, Istituto Italiano di Tecnologia, 00161, Rome, Italy; Neuroscience and Behaviour Laboratory, Istituto Italiano di Tecnologia, 00161, Rome, Italy; Department of Neuroscience, Physiology and Pharmacology, University College London (UCL), WC1E 6BT, London, UK

**Keywords:** behavioral relevance, Electroencephalography (EEG), stimulus offset, surprise, Vertex Potential

## Abstract

Abrupt increases of sensory input (*onsets*) likely reflect the occurrence of novel events or objects in the environment, potentially requiring immediate behavioral responses. Accordingly, *onsets* elicit a transient and widespread modulation of ongoing electrocortical activity: the Vertex Potential (VP), which is likely related to the optimisation of rapid behavioral responses. In contrast, the functional significance of the brain response elicited by abrupt decreases of sensory input (*offsets*) is more elusive, and a detailed comparison of *onset* and *offset* VPs is lacking. In four experiments conducted on 44 humans, we observed that *onset* and *offset* VPs share several phenomenological and functional properties: they (1) have highly similar scalp topographies across time, (2) are both largely comprised of supramodal neural activity, (3) are both highly sensitive to surprise and (4) co-occur with similar modulations of ongoing motor output. These results demonstrate that the *onset* and *offset* VPs largely reflect the activity of a common supramodal brain network, likely consequent to the activation of the extralemniscal sensory system which runs in parallel with core sensory pathways. The transient activation of this system has clear implications in optimizing the behavioral responses to surprising environmental changes.

## Introduction

To survive in a rapidly changing world, animal brains have evolved the ability to build expectations about the sensory environment. Sudden environmental events that violate these expectations have a clear importance to survival, as they might require a rapid behavioral response. Indeed, failing to respond to them appropriately could result in capture by a predator, injury by environmental dangers, or a missed opportunity to catch a prey. Perhaps the simplest examples of such events are abrupt and unexpected increases or decreases of sensory intensity (referred to as *onsets* and *offsets* from here onward), which violate the most basic assumption that the sensory input will not rapidly deviate from the immediately preceding *status quo*.

The brain response to sudden *onsets* has been extensively studied: when neural activity is measured using scalp electroencephalography (EEG), it consists in a transient and extremely large electrocortical biphasic wave spread across much of the scalp, equivalent to the response elicited by impulse stimuli (Nishihara et al. 2011; Somervail et al. 2021) (therefore, we use the term *onset* to refer also to impulse stimuli). Being maximal at the scalp vertex, this wave is known as the Vertex Potential (VP) or Vertex Wave, and consists of a negative–positive (N-P) complex in the time-domain (Bancaud et al. 1953; Mouraux and Iannetti 2009). Both the sensitivity to environmental features and the functional significance of this response have been extensively studied. For example, the VP magnitude is highly dependent on the degree of *unexpectedness* or *surprise* of the eliciting stimulus, which reflects the degree to which it stands out from recent or neighboring sensory input. This surprise is determined by at least two different kinds of change (Näätänen and Picton 1987): the degree to which the *onset* stands out from the immediately preceding baseline level (i.e., the differential intensity of the *onset*; Somervail et al. 2021) and the degree to which the *onset* differs with respect to a stream of previously occurring *onsets* (Iannetti et al. 2008; Wang et al. 2010; Valentini et al. 2011; Ronga et al. 2013). In contrast to the high sensitivity to unexpectedness, the VP is largely insensitive to the modality of the eliciting stimulus: VPs highly similar in shape and magnitude can be elicited by stimuli of different modalities, provided that they are equally salient (Mouraux and Iannetti 2009; Kilintari et al. 2018). Accordingly, blind source separation and source analysis of VPs elicited by stimuli of different sensory modalities (e.g., audition, vision, somatosensation) revealed that most of the variance comprising the VP reflects supramodal neural activity (Mouraux and Iannetti 2009; Liang et al. 2010). With respect to the functional significance of the VP, we and others have recently demonstrated that it is not a mere sensory phenomenon, but a motoric one: indeed, it is tightly coupled with a multipolar modulation of muscular activity (Novembre et al. 2018, 2019), and it predicts the latency of speeded reaction times (Moayedi et al. 2015; Tiemann et al. 2018), providing evidence for an active role in urgent behavior.

In contrast to this wealth of knowledge, the brain responses to abrupt and unexpected *offsets* have been investigated far less. The imbalance between studies of neural responses to *onsets* and *offsets* is surprising, given that *offsets* can also reflect environmental events demanding swift and potentially life-saving behavioral responses: for example, the sudden dimming of light intensity can reflect a predating hawk (and in fact triggers freezing behavior in chicks; Hébert et al. 2019). Accordingly, one might hypothesize that the brain responses to both *onsets* and *offsets* reflect the functioning of a common neural system devoted to the detection of, and appropriate reaction to, abrupt intensity changes *of any kind* (i.e., regardless of their direction or the sensory modality in which they occur).

Unsurprisingly, a few studies have indeed shown that abrupt *offsets* of both auditory and somatosensory stimuli also elicit a negative–positive EEG potential, maximal at scalp vertex and qualitatively similar to the VP elicited by *onsets*, although typically smaller in magnitude (Davis 1939; Davis and Zerlin 1966; Onishi and Davis 1968; Schweitzer and Tepas 1974; Schweitzer 1977; Parker et al. 1982; Jones 1992; Yamashiro et al. 2008; Baltzell and Billings 2014). All these studies, however, present several fundamental issues related to their experimental design, data analysis, and result interpretation.

First, experimental designs were often unsuitable to obtain a fair comparison of *onset*- and *offset*-evoked VPs, as *onset* stimuli generally occurred at relatively long or more variable time after the previous *offset* stimulus (e.g., 10–12 s; Yamashiro et al. 2008), whereas *offset* stimuli often followed more predictably and/or sooner after the preceding *onset* (typically by less than 3 s; e.g., Yamashiro et al. 2008). Given the well-known dependence of VP amplitude on the temporal predictability of the eliciting stimulus (e.g., Iannetti et al. 2008), it is not surprising that these designs resulted in habituated *offset* VPs of smaller amplitude than *onset* VPs (Davis 1939; Onishi and Davis 1968; Spychala et al. 1969; Schweitzer and Tepas 1974; Schweitzer 1977; Parker et al. 1982; Spackman et al. 2006; Yamashiro et al. 2008). This habituation, consequent to imperfect experimental paradigms, prevents an adequate comparison of several response features. For example, the habituation of some response subcomponents (but not others) could alter the overall scalp distribution, and thereby prevent adequate spatial comparison of the *onset* and *offset* VPs. Additionally, this same habituation could obscure possible behavioral consequences of the *offset* VP, such as the modulations of motor output elicited by impulse stimuli (Novembre et al. 2018).

Second, a proper quantitative comparison of the evolution of the scalp distributions of *onset* and *offset* responses across time was missing. This is largely due to the historical use of low-density EEG systems unable to adequately capture the response scalp distribution (Davis 1939; Onishi and Davis 1968; Spychala et al. 1969; Schweitzer and Tepas 1974; Elfner et al. 1976; Schweitzer 1977; Hillyard and Picton 1978; Parker et al. 1982; Jones 1992; Yamashiro et al. 2008), and also to the habit, widely accepted until the 90s, to only measure the peak amplitude of the main VP waves (Davis and Zerlin 1966; Onishi and Davis 1968; Spychala et al. 1969; Schweitzer and Tepas 1974; Schweitzer 1977; Hillyard and Picton 1978; Parker et al. 1982; Jones 1992; Spackman et al. 2006; Yamashiro et al. 2008; Baltzell and Billings 2014).

Third, several authors have too quickly assumed that *offset* VPs reflect modality-specific sensory systems (Spychala et al. 1969; Jones 1992; Spackman et al. 2006; Baltzell and Billings 2014). For example, VPs elicited by auditory *offsets* are often explicitly interpreted as reflecting the functioning of the auditory system (e.g., for sound perception), without considering the possibility that the responses are instead supramodal (Jones 1992; Baltzell and Billings 2014). Even when not stated explicitly, this interpretation is implied due to the focus of the authors on a single sensory system, such as the auditory system (Schweitzer and Tepas 1974; Elfner et al. 1976; Schweitzer 1977). As such, the functional properties of these responses have usually not been interpreted beyond the realm of perception within single sensory modalities. Notably, the literature describing evoked potentials to *onset* stimuli is not devoid of this fundamental problem either. For a review on the topic, see Mouraux and Iannetti (2018).

Consequently, whether the VPs elicited by abrupt *offsets* reflect the activity of the same supramodal neural system activated by *onsets* remains an unanswered question. Without such basic knowledge, our understanding of the functional significance of these large brain responses remains incomplete. In the current set of experiments, we tackled this issue by recording brain activity with 64-channel EEG (i.e., at higher density than previous studies), using stimulation paradigms specifically designed to allow a fair comparison of both the phenomenological and functional properties of *onset* and *offset* responses. Should *onset* and *offset* responses reflect the functioning of the same neural system, we predicted that they would (1) have quantitatively highly-similar temporal evolution of their scalp distributions, and (2) be largely composed of similar, supramodal components. We also predicted that, like their *onset*-evoked counterpart, *offset*-evoked VPs would (3) be highly sensitive to the unexpectedness of the eliciting stimulus, and (4) co-occur with similar activations of the motor system. In four Experiments conducted on 44 healthy human participants we thoroughly tested these predictions.

## Materials and Methods

### Participants

A total of 34 unique healthy human participants (29 males, mean ± SD age, 31 ± 10 years, age range 19–72 years) took part in one or more out of four experiments (N = 14, 10, 14 and 20; Exp 1, 2, 3 and 4 respectively). All participants gave written informed consent before taking part in the study. All procedures were approved by the local ethical committee.

### Sensory Stimulation

In all four experiments, participants received either auditory or somatosensory (mechanical) tonic stimuli. In Experiments 1, 3 and 4, participants received auditory stimuli, consisting of 600 Hz pure tones delivered binaurally through pneumatic insert-earphones (Etymotic ER-3C 10 Ohm). In Experiment 2, participants received the same auditory stimuli, but delivered through a loudspeaker (Q Acoustics 3020), as well as tonic, non-painful mechanical stimulation on the right-hand dorsum. Mechanical stimulation was delivered manually by the experimenter using a cylindrical stainless-steel wire with a flat tip (diameter = 0.25 mm), mounted on a plastic rod with a weight, which was free to move inside a handheld stainless-steel tube (Iannetti et al. 2013). Consequently, when the rod was applied perpendicularly to the skin, it exerted a constant force of ~ 128 mN. Precise timing of mechanical stimulation was measured by connecting a 1.5 V battery to the stimulator and stimulation site to create an electric circuit upon contact with the skin; the resulting potential difference was measured by two electrodes, one placed on the hand near the stimulation site and the other placed on the upper arm. We did not use electrical stimulation for the somatosensory stimulus given that, when delivered through pulses repeated at high frequency, it elicits a train of distinct perceptual events, which hardly fuse into a smooth, constant sensation as does the mechanical stimulation we chose. In all experiments, auditory stimulation was controlled using MATLAB (Mathworks) and the Psychophysics toolbox (Brainard 1997). Accurate timing of somatosensory stimulation in Experiment 2 was ensured by playing through headphones the same auditory stimuli to the experimenter delivering the mechanical stimuli.

### Experimental Design

All experiments were conducted in a dim, silent, temperature-controlled room. During EEG recording, participants were required to keep their gaze on a fixation cross (4 x 4 cm) placed centrally in front of them, at approximately 30° below eye-level. Between blocks, participants were allowed to relax for up to 2 minutes.

**Figure 1:**
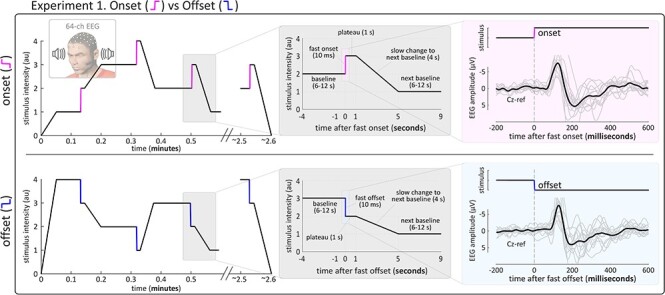
Experiment 1: stimulation profile and experimental design. *Left.* Stimulation profile of typical *onset* (top) and *offset* (bottom) blocks in Experiment 1. Stimulus intensity abruptly increased (*onset*; pink segments) or decreased (*offset*; blue segments) from a baseline level to a target level in 10 ms (colored segments), plateaued for 1 s and then slowly increased or decreased to the next baseline level in 4 s. All abrupt intensity changes had the same differential intensity (1/4 of the intensity range). *Right.* Grand averages (black) and participant-level averages (gray) elicited by *onsets* (top) and *offsets* (bottom).

In Experiment 1, abrupt *onsets* and *offsets* of stimulus intensity (rise/fall time = 10 ms) were delivered in separate blocks (Fig. 1). In each *onset* or *offset* the difference between baseline and target intensity (i.e., the differential intensity) was identical. Figure 1 shows the stimulation profiles of representative blocks of *onsets* and *offsets*: before each abrupt change, the baseline intensity level was reached by slowly changing the intensity from the target level of the previous change (4 s). After each abrupt change, stimulus intensity remained at target level for 1 s. The mean interval between two consecutive changes (i.e., between two trials) was 14 s (11–17 s; uniform distribution). Each participant received 12 blocks of stimuli, each lasting ~ 2.5 mins and containing 12 abrupt changes, yielding 144 changes in total (72 *onsets* and 72 *offsets*). *Onset* and *offset* blocks were delivered in pseudorandom order, with the constraint that no block of the same type was repeated more than twice in a row.

In Experiment 2, participants received tonic auditory and somatosensory stimuli in separate blocks. In each block, abrupt *onsets* and *offsets* of stimulus intensity (auditory rise/fall time = 10 ms) were embedded in the stimulation profile. Participants sat in front of a table with their stimulated (right) hand resting on the table surface, while the experimenter sat on the opposite side, facing the participant. A curtain prevented participants from seeing both the stimulated hand and experimenter. The loudspeaker delivering the auditory stimuli was placed near the stimulated hand. During EEG recording, the intensity of the ongoing stimulus would abruptly increase (*onset*), remain at a peak intensity level for 8–14 s (uniform distribution), and then abruptly decrease (*offset*) and remain at zero intensity for 8–14 s before the next *onset*. Thus, *onsets* and *offsets* were delivered in a continuous stream, and were preceded and followed by the next *onset* or *offset* after a variable and unpredictable interval. Auditory and somatosensory stimuli were delivered in 8 alternating blocks (balanced across participants). Each block lasted ~ 2.2 mins and contained 12 abrupt changes, yielding 96 changes in the entire experiment (24 *onsets* and *offsets* for each sensory modality). The intensity level of auditory and somatosensory stimuli was carefully matched in each subject, in a preliminary session using the following procedure. The intensity of the mechanical stimulus was constant and determined by the force elicited by the stimulator (~128 mN), and was therefore used as an anchor. These mechanical stimuli were delivered for approximately 5 seconds and alternated with auditory stimuli also delivered for 5 seconds. Participant were required to verbally report whether each auditory stimulus was more or less intense than the preceding mechanical stimulus. The volume of the following auditory stimulus was accordingly increased or decreased by 3 dB. This procedure continued until the participant reliably reported that the two stimuli elicited sensations of similar intensity.

In Experiment 3, three consecutive auditory changes (rise/fall time = 10 ms) of identical differential intensity were repeated at a frequency of 1 Hz (a triplet: S1-S2-S3; Iannetti et al. 2008). *Onsets* and *offsets* were never intermixed within the same triplet. Before each triplet, the baseline level preceding the first change (S1) was reached by slowly modulating the intensity level (duration: 4 s) from the target level of the last change (S3) of the previous triplet. The mean interval between two consecutive triplets (e.g., from the S1 of a given triplet to the S1 of the following triplet) was 16 s (13–19 s; uniform distribution). Each participant received 4 blocks of stimulation. Each block lasted ~ 3 minutes and contained 12 triplets, yielding 48 triplets in the entire experiment (24 triplets for each of the two conditions).

In Experiment 4, participants were required to perform a simple motor task, in which they exerted a constant force (~1.5 N) on an isometric force transducer held between their index finger and thumb (Novembre et al. 2018, 2019). At the beginning of each block, participants were instructed to exert a gradually increasing force while receiving verbal feedback about the force applied: once a force level between 1.25 and 1.75 N was reached, participants were instructed to keep the force applied as constant as possible, and at that point the recording started. Throughout the recording block, while performing the motor task, participants received task-irrelevant auditory stimuli with embedded abrupt changes (rise/fall time = 5 ms). The stimulation profile was similar to Experiment 1, except for the following three differences: (1) *onsets* and *offsets* were intermixed within each block (pseudorandomised with the constraint that no more than 3 consecutive intensity changes could have the same direction); (2) the plateau following each change lasted 3 s instead of 1 s, to allow better sampling of the stimulus-induced force modulation, which can last up to 3 s (Novembre et al. 2018, 2019); and (3) stimulus intensity always increased to and decreased from the same peak intensity (as in Experiment 2), which was set before the experiment to the highest intensity the participant could tolerate. Each participant received 6 blocks of stimuli. Each block lasted ~ 2.5 mins and contained 10 abrupt changes, yielding 60 changes in the entire experiment (30 *onsets* and 30 *offsets*).

### E‌EG Recording and Preprocessing

In all experiments, the electroencephalogram (EEG) was recorded using 64 active electrodes placed on the scalp according to the International 10–10 system and referenced to the nose. EEG signals were amplified and digitized using a sampling rate of 2048 Hz (Biosemi Active-2 system), then preprocessed and analyzed using MATLAB (version 2018a, MathWorks), Letswave (Mouraux and Iannetti 2008), and Fieldtrip (Oostenveld et al. 2011). Continuous EEG data were first band-pass filtered between 0.5 and 30 Hz (Butterworth). Data were then segmented into 4-s long epochs (−2 to +2 s relative to the beginning of each abrupt intensity change). Artifacts due to eye blinks or eye movements were removed using a validated method based on independent component analysis (Jung et al. 2000). Within each epoch, any electrode with amplitude values exceeding ±100 μV was interpolated by averaging the signal sampled from its neighboring electrodes; if more than three electrodes needed interpolation, the epoch was rejected. Remaining epochs were baseline corrected (reference interval − 0.2 s to 0 s). The average percentage of rejected epochs per participant was (mean ± std): 3.5 ± 3.4% [Experiment 1], 5.4 ± 4.9% [Experiment 2], 3.6 ± 4.6% [Experiment 3], and 3.6 ± 5.4% [Experiment 4]. Finally, average ERP waveforms were computed for each participant and condition.

### Force Recording and Preprocessing

The force applied by participants in Experiment 4 was sampled at 1000 Hz using a force-torque transducer (ATI nano17, Industrial Automation) and custom software written in LabVIEW (National Instruments). At the start of each recording session, the force value was set to zero to mitigate the effects of slow sensor drifts. To facilitate the two-finger grip, the transducer was mounted between two cylindrical plastic extensions. Continuous data were segmented using a time-window from −0.4 to 3 s relative to the beginning of each abrupt intensity change (epoch duration = 3.4 s). Epochs contaminated by artifacts (deviating, at any timepoint, more than 3 SDs from the participant’s mean exerted force across all trials) were excluded from further analysis. The corresponding EEG epochs were also excluded. Consequently, the percentage of rejected epochs was the same as the EEG data: 3.6 ± 5.4%. Finally, epochs were baseline corrected using the −0.05 to 0 s prestimulus interval, and high-pass filtered to isolate the transient force modulations (Novembre et al. 2018).

### Statistical Analysis

In Experiment 1, we compared the scalp distribution of the ERPs elicited by *onsets* and *offsets* by calculating the spatial correlation (i.e., the correlation across channels) between the average waveforms for each condition, for each timepoint and each participant (Murray et al. 2008). The across-subject consistency of spatial correlation timecourses was statistically assessed by performing a point-by-point one-sample t-test (against zero) of the (Fisher’s z-transformed) spatial correlation values of each participant, with cluster permutation testing (1000 permutations; Maris and Oostenveld 2007).

In Experiment 2, we explored the selectivity of the constituent components of the ERPs elicited by abrupt *onsets* and *offsets* of both auditory and somatosensory stimuli. We first cropped the participant-level average waveforms for each of the four conditions between −0.5 and + 1.5 s and concatenated them. We then decomposed the waveforms into a set of independent components (ICs) of fixed scalp topography using probabilistic independent component analysis (pICA; Beckmann and Smith 2004; Mouraux and Iannetti 2008, 2009). pICA uses an estimate of the intrinsic dimensionality of the data to approximate the true number of independent sources contributing to the signal. As a result, each IC is more likely to reflect a single physiological source of activity compared to a traditional unconstrained ICA (Mouraux and Iannetti 2008). We then computed, for each IC, the proportion of signal variance explained at each timepoint by dividing their global field power by the total global field power across all ICs. These proportions were subsequently averaged across the post-stimulus interval (0 to +0.5 s) separately for each condition, yielding four values for each IC, reflecting the mean explained variance for each condition. To quantify how selective the ICs were for each of the four conditions, we then calculated the correlation (Pearson, r) between these explained variance values for each pair of conditions across all ICs (i.e., at group-level). As a summary value of the selectivity of each IC, we computed a selectivity ratio which was equal to the largest explained variance value divided by the mean explained variance across the rest of the conditions—this value therefore reflected how selectively the IC explained variance for one condition. We then correlated (Spearman’s rank, r_s_) these selectivity ratios with the mean variance explained in all conditions, across all ICs.

In Experiment 3, we compared the ERPs elicited by each of the three stimuli composing the triplet (S1-S2-S3), separately for *onset* and *offset* triplets. Participant-level averages for each condition were analyzed using a point-by-point, two-way repeated-measures ANOVA in the time-window −0.2 to 0.6 s in each channel, with factors “change direction” (two levels: onset and offset) and “stimulus repetition” (three levels: S1, S2 and S3). Cluster permutation testing was used to correct for multiple comparisons (1000 permutations).

In Experiment 4, we analyzed single-subject average force waveforms using point-by-point, one-sampled t-tests against zero (i.e., against the mean baseline amplitude), to determine the response consistency across participants. Cluster permutation testing was used to correct for multiple comparisons (1000 permutations).

## Results

### Experiment 1. Auditory Onsets and Offsets Elicit Highly Similar Vertex Potentials (Prediction 1)

**Figure 2:**
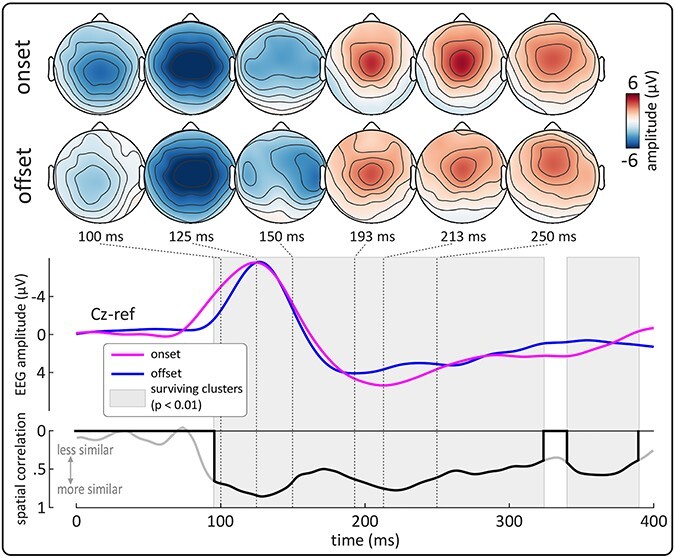
Experiment 1: abrupt *onsets* and *offsets* of auditory stimuli elicit highly-similar Vertex Potentials. Topographies show the evolution of the scalp distribution of *onset* and *offset* ERPs over time. The top plot shows the grand-average waveforms (Cz) elicited by abrupt auditory *onsets* and *offsets*. The bottom plot shows the timecourse of the mean spatial correlation between the two waveforms. Gray areas show time intervals in which spatial correlation was statistically significant at group-level. Both responses had highly similar scalp distributions throughout their timecourse. The similarity was strongest at the peak latencies, where both responses were dominated by widespread negative and positive waves, maximal at scalp vertex (Vertex Potentials).

In Experiment 1, we compared the spatial distribution of the brain responses elicited by increases (*onsets*) and decreases (*offsets*) of stimulus intensity with equal differential intensity and equal rise or decay time, embedded within an ongoing auditory stimulus (Fig. 1, left panel). Figures 1 and 2 show the single-subject and group-level average waveforms elicited by *onsets* and *offsets*. Morphology and topography of the responses were qualitatively similar: both *onsets* and *offsets* elicited a large, widespread negative–positive (N-P) complex, maximal at the scalp vertex (Cz) and peaking at approximately 124 and 127 ms (N wave, *onset* and *offset* condition respectively), and 193 and 213 ms (P wave, *onset* and *offset* condition respectively) (group-level average waveforms, Figures 1 and 2).

To quantitatively compare the temporal evolution of the two responses across the scalp, we computed the spatial correlation (Murray et al. 2008) between the participant-level average *onset* and *offset* waveforms, for each condition at each timepoint (Fig. 2). We observed strong evidence that the spatial distributions of *onset* and *offset* responses were very similar in a large post-stimulus interval (84–330 ms; cluster p < 0.01). Spatial correlations were overall strong and maximal at approximately 130 and 220 ms, i.e., around the peak latencies of the N and P waves in the grand average waveform (mean r = 0.85 and 0.77 for N and P waves, respectively).

### Experiment 2. Offset-evoked Vertex Potentials are highly Supramodal (Prediction 2)

**Figure 3:**
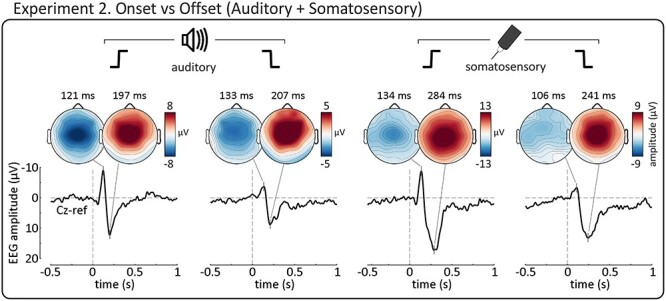
Experiment 2: both *onset* and *offset* Vertex Potentials are highly supramodal. Plots show the grand-average waveforms (Cz) elicited by abrupt auditory *onsets* (far-left), auditory *offsets* (middle-left), somatosensory *onsets* (middle-right) and somatosensory *offsets* (far-right). Scalp distributions are shown for the N and P peak of each ERP. All four waveforms were dominated by highly similar Vertex Potentials, although the N wave of the somatosensory *offset* VP overlapped with a left-lateralised component, possibly reflecting the activity of the primary somatosensory cortex contralateral to the stimulated hand (Valentini et al. 2012).

In Experiment 2, we employed a novel 2x2 experimental design to compare the VPs elicited by *onsets* and *offsets* in two sensory modalities: somatosensation and audition. This design not only allowed us to test Prediction 2 (that, like *onset-*evoked VPs, *offset*-evoked VPs would largely reflect supramodal neural activity), but also provided further evidence that Prediction 1 was correct, in a different group of participants and across two modalities. Figure 3 shows the group-level average waveforms of Experiment 2. As in Experiment 1, both *onsets* and *offsets* elicited highly similar negative–positive complexes maximal at scalp vertex. One minor exception was that the N wave elicited by somatosensory *offsets* had a less central scalp distribution, seemingly because a left-lateralised subcomponent, possibly reflecting the primary somatosensory cortex contralateral to the stimulated hand (Valentini et al. 2012), was more visible given the smaller overlapping N wave of the *offset* Vertex Potential.

**Figure 4:**
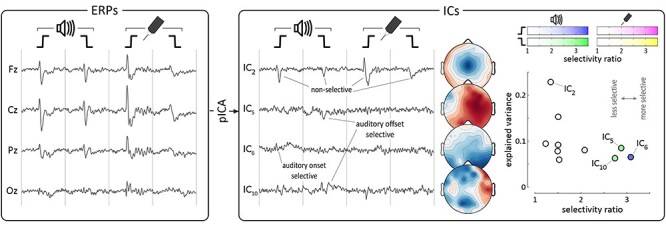
Experiment 2: probabilistic independent component analysis (pICA) applied to the average waveforms of an example participant. We used pICA to decompose the concatenated participant-level averages (left panel) into a set of temporally-independent and spatially-fixed independent components (ICs) best reflecting the data (right panel). Four example ICs are shown along with their spatial distributions. The scatterplot shows how selective each component was for a particular condition, compared with how much variance it explained on average. Color opacity reflects the selectivity for a particular condition (auditory *onset*: blue; *offset*: green; somatosensory *onset*: pink; *offset*: yellow). The three most selective components (IC 5, 6 and 10) were somewhat selective for auditory *offset* (green), *onset* (blue) and *offset* respectively. Note that the largest component (IC 2) was highly unselective, while the most selective components did not contribute greatly to the overall variance of the waveforms.

To quantitatively determine the condition-wise selectivity of the neural activity underlying these responses (and thereby test Prediction 2), we first concatenated the participant-level averages across the four experimental conditions (auditory *onset*, auditory *offset*, somatosensory *onset*, somatosensory *offset*). We then decomposed these waveforms into their underlying components using probabilistic independent component analysis (pICA). In contrast to standard ICA, where the number of independent components (ICs) is either equal to the number of recording channels or has to be defined manually *a priori*, pICA estimates the true number of ICs from the data (Beckmann and Smith 2004; Mouraux and Iannetti 2009; see Methods). This approach is outlined in Figure 4, using data from an example participant.

**Figure 5:**
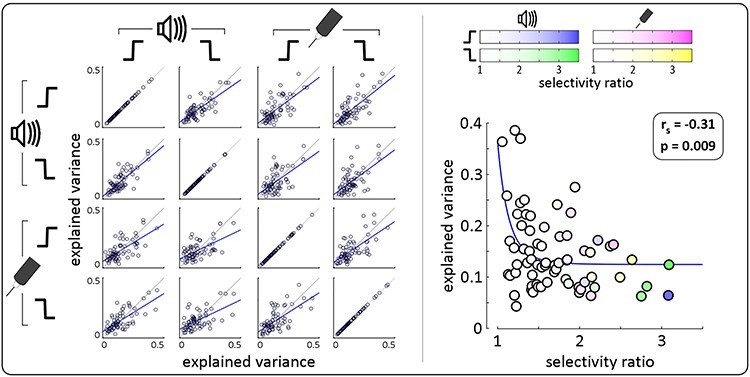
Experiment 2: group-level pICA results. Supramodal, non-specific components explained the most variance. *Left*. Scatterplots show, for each component, the mean explained variance in each pair of conditions, at group-level (i.e., circles show components from each participant). Blue lines show linear regression. Gray lines are identity lines. Strong positive correlations can be seen in all scatterplots, showing that components explaining a certain amount of variance in one stimulus condition were likely to explain a similar amount of variance in other conditions. *Right*. The scatterplot shows how selective these same components were for a particular condition, compared with how much variance they explained on average. Color opacity indexes the selectivity for a particular condition (auditory *onset*: blue; *offset*: green; somatosensory *onset*: pink; *offset*: yellow). Blue line shows non-linear regression (power law). The strong negative correlation shows that components explaining the most variance were also the least selective, while the most selective components explained the least variance.

To quantify the degree of selectivity of the resulting ICs for each of the four conditions, we first computed the mean variance explained by each IC for each condition (0 to +0.5 s post-stimulus), and then calculated their correlations in all possible pairs of experimental conditions and across all ICs (i.e., at group-level). All correlations (Fig. 5, left panel; see Table 1 for r and p values) were strong and positive, indicating that ICs explaining a certain degree of variance in one condition were very likely to explain a similar degree of variance in the other conditions. In other words, there were no or few ICs explaining a large degree of variance in only one condition.

**Table 1:** Correlations of explained variance between each condition, across components

***r values***		**auditory**		**somatosensory**	
		***onset***	***offset***	***onset***	***offset***
**auditory**	***onset***	**n/a**	**0.77**	**0.69**	**0.72**
	***offset***	**0.77**	**n/a**	**0.59**	**0.60**
**somatosensory**	***onset***	**0.69**	**0.59**	**n/a**	**0.67**
	***offset***	**0.72**	**0.60**	**0.67**	**n/a**
					
***p values***		**auditory**		**somatosensory**	
		***onset***	***offset***	***onset***	***offset***
**auditory**	***onset***	**n/a**	**9.3E-15**	**6.4E-11**	**4.7E-12**
	***offset***	**9.3E-15**	**n/a**	**1.0E-07**	**4.6E-08**
**somatosensory**	***onset***	**6.4E-11**	**1.0E-07**	**n/a**	**4.0E-10**
	***offset***	**4.7E-12**	**4.6E-08**	**4.0E-10**	**n/a**

As a summary value of the selectivity of each IC, we computed the ratios of explained variance across conditions (see Methods for details)—the larger the ratio the more selective the IC. The key result is that the selectivity ratio was highly negatively correlated with the mean explained variance across all conditions (r_s_ = −0.31, p = 0.009; Fig. 5, right panel). This indicates that non-direction- and non-modality- selective ICs (i.e., ICs explaining both *onset* and *offset* responses, in both somatosensory and auditory conditions) reflected more of the neural activity underlying the responses than the more selective ICs. Altogether, these results show that the brain responses observed in each of the four conditions were dominated by similar neural activity, which was highly supramodal and non-specific for either *onsets* or *offsets*.

### Experiment 3. Both Onset- and Offset-evoked Vertex Potentials are Highly Sensitive to Stimulus Surprise (Prediction 3)

The results of Experiments 1 and 2 show substantial phenomenological and compositional similarity between the responses elicited by *onsets* and *offsets*, regardless of whether the eliciting stimulus was auditory or somatosensory. In Experiment 3 we expanded on these findings by exploring the sensitivity of the responses to the unexpectedness or surprise content of the eliciting stimulus. It is well-established that *onset*-evoked VPs are highly sensitive to the surprise content of the eliciting stimulus, with more surprising stimuli producing a VP of larger amplitude (Wang et al. 2010; Valentini et al. 2011; Ronga et al. 2013). Should the VPs elicited by abrupt *offsets* reflect the same neural system subserving *onset-*evoked VPs, it follows that *offset* responses should also be highly sensitive to this factor.

**Figure 6:**
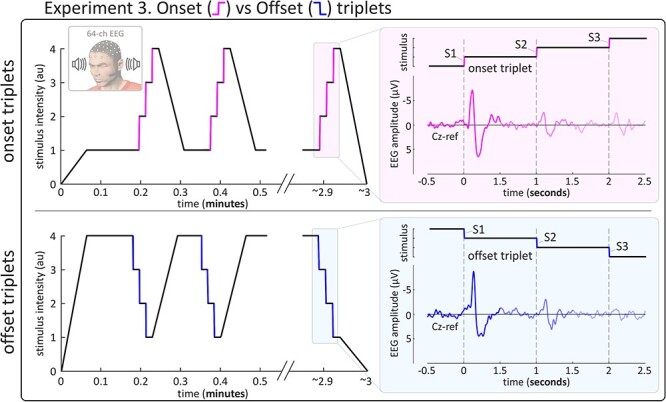
Experiment 3: stimulation profile and experimental design. *Left.* Stimulation profile of typical *onset* (top) and *offset* (bottom) blocks in Experiment 3. From baseline, stimulus intensity abruptly increased (*onset*) or decreased (*offset*) three times in a row (S1-S2-S3) with a 1-s interval between each change (i.e., a triplet at 1 Hz). Before each triplet, the baseline level preceding the first change (S1) was reached by slowly changing the intensity level from the previous triplet in 4 s. *Right.* Grand averages for the Vertex Potentials (VPs) elicited by the three stimuli in the triplet. Repetition of the abrupt change reduced the magnitude of subsequent VPs, for both *onsets* and *offsets*.

To test this hypothesis, we exploited an established paradigm that effectively dissociates the magnitude of the afferent sensory barrage from its surprise content by modulating temporal predictability: we delivered a train of three consecutive changes (i.e., a triplet: S1, S2, S3) of either *onsets* or *offsets* with identical differential intensity (Somervail et al. 2021), at 1 Hz (Fig. 6). In this paradigm, S2 and S3 are more temporally predictable than S1 and therefore less surprising (Iannetti et al. 2008).

**Figure 7:**
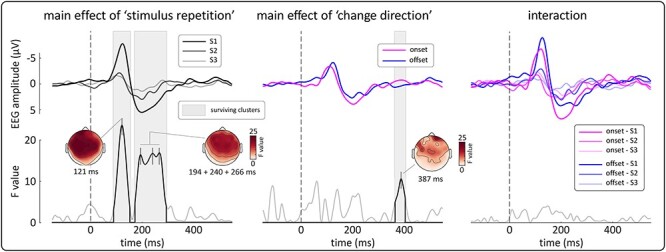
Experiment 3: o*nset*- and *offset*-evoked potentials similarly habituated when the stimulus was repeated predictably at 1 Hz. *Top row.* Group-level average waveforms (Cz) for each level of “stimulus repetition” (S1, S2, S3; left), “change direction” (onset, offset; middle) and for each individual condition (right). *Bottom row.* F-value timecourses for each factor (Cz). Gray areas show significant clusters after permutation testing. The N and P waves were both significantly modulated by factor “stimulus repetition”, reflecting the habituation of the Vertex Potentials (VPs) after the first abrupt change (S1). Importantly, these effects were widespread across the scalp and there was no evidence of an interaction, indicating that the *onset* and *offset* VPs habituated similarly across the scalp. There were no significant effects associated with the factor “change direction” during the timecourse of the VP. These results show that that similar underlying neural generators were modulated by stimulus repetition and provide further evidence that the *onset* and *offset* VPs reflect a common brain network.

In both *onset* and *offset* triplets, stimulus repetition resulted in a clear reduction of VP amplitude: S2 and S3 amplitudes were lower than the amplitude of S1 (Fig. 6, right). These observations, which dovetail previous findings using *onset* stimuli (Ritter et al. 1968; Iannetti et al. 2008; Wang et al. 2010; Valentini et al. 2011; Liberati et al. 2018), were substantiated by a two-way ANOVA with factors: “change direction” (two levels: onset and offset), and “stimulus repetition” (three levels: S1, S2 and S3). Figure 7 shows the results of this ANOVA. There was strong evidence of a main effect of “stimulus repetition” between ~ 89–150 ms and ~ 168–297 ms (cluster p = 0.006, in both intervals), i.e., around the peak latency of the main vertex waves. Importantly, the scalp distribution of these main effects was widespread (Fig. 7), and there was no evidence of a “change direction” x “stimulus repetition” interaction. These two results indicate that the spatial distribution of the surprise-dependent habituation of the VP was similar across the *onset* and *offset* conditions. Finally, there was no evidence of a main effect of “change direction” until well after the VP latency (at ~ 390 ms). Overall, these three results suggest that similar constituent components were habituated by stimulus repetition, thus providing further evidence that the VPs elicited by *onsets* and *offsets* reflect a common underlying network sensitive to stimulus surprise.

### Experiment 4. Onset and Offset Vertex Potentials Co-occur with Similar Modulations of Motor Output (Prediction 4)

The results of Experiments 1–3 provide strong evidence that the VPs evoked by *onsets* and *offsets* reflect the functioning of a common neural system. A final important question is whether *onsets* and *offsets* are similarly related to behavior. Our group has recently demonstrated that VPs elicited by *onset* stimuli are tightly coupled with a modulation of muscular output during an isometric force task (Novembre et al. 2018, 2019).

**Figure 8:**
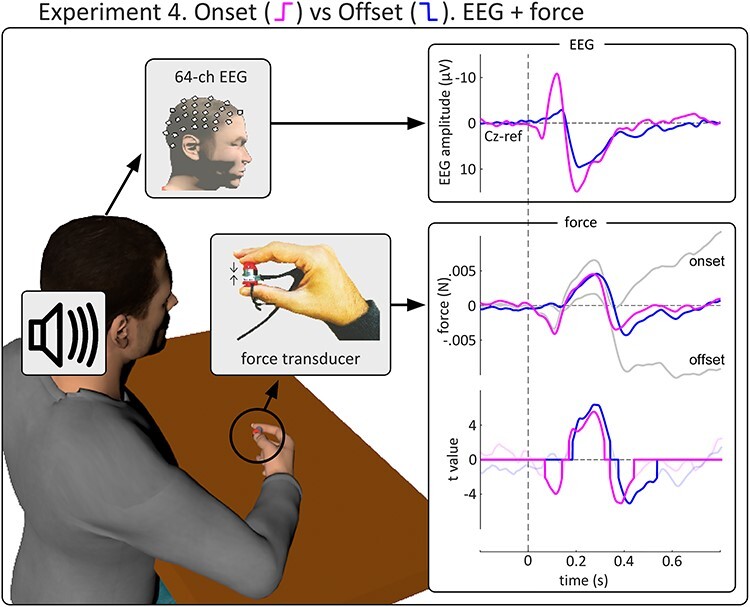
Experiment 4: abrupt *onsets* and *offsets* elicit similar modulations of motor output during an isometric force task. *Left.* Experimental setup of Experiment 4: participants sat at a table applying a constant force with their index and thumb (measured by a force transducer), while receiving abrupt auditory *onsets* and *offsets*. *Right.* Top row shows the grand-average EEG responses elicited by *onsets* (pink) and *offsets* (blue). Middle row shows the grand-average force modulations. Colored plots show the high-pass filtered signals; gray plots show the unfiltered signals. Bottom row shows the t-value timecourse from the t-tests against zero across participants. Opaque lines show significant clusters. *Onsets* and *offsets* both elicited a similar transient increase of force at ~ 280 ms, followed by a decrease at ~ 400 ms. *Onsets*, but not *offsets*, elicited an initial force decrease at ~ 100 ms. These results indicate that both *onsets* and *offsets* elicited a largely similar multiphasic pattern of force modulations. Unfiltered force plots (in gray, bottom right panel) show that both *onsets* and *offsets* both elicited a late force modulation, albeit in the opposite direction.

In Experiment 4, we tested the motor consequences of stimulus *offsets*. We used a highly-sensitive force transducer to record fine variations in the isometric force exerted by participants. Both *onsets* and *offsets* elicited a transient and multipolar force modulation, similar to that previously observed in response to *onset* stimuli (Figure 8, middle). *Onsets* elicited an initial force decrease at ~ 110 ms, followed by a force increase at ~ 270 ms and a further decrease at ~ 370 ms (Fig. 8; Novembre et al. 2018). *Offsets* elicited a similar increase and decrease of force at ~ 280 and ~ 410 ms, respectively, although with no initial decrease (perhaps related to the lack of a clear early deflection or the smaller N wave in the corresponding EEG response; Fig. 8). These observations were substantiated with point-by-point t-tests against zero (Fig. 8, bottom). Altogether, these results show that the *onset* and *offset* VPs co-occur with similar modulations of motor output.

We finally note that before applying the high-pass filter necessary to highlight the transient force modulations (Novembre et al. 2018), a long-latency and long-lasting force modulation was present for both *onsets* and *offsets* (Fig. 8, gray waveforms). Interestingly, the polarity of this force modulation was opposite in the two conditions: positive when elicited by *onsets* and negative when elicited by *offsets*—a finding possibly hinting towards a differential effect of change direction on delayed behavior, clearly deserving further investigation.

## Discussion

In this study, we compared the EEG response elicited by abrupt and unexpected stimulus *offsets* with the well-characterized Vertex Potentials (VPs) elicited by stimulus *onsets.* Previous studies have highlighted the importance of *onset*-evoked VPs, showing that they reflect a neural system highly sensitive to surprising and therefore behaviorally-relevant environmental changes (Iannetti et al. 2008; Wang et al. 2010; Valentini et al. 2011; Torta et al. 2012; Ronga et al. 2013; Somervail et al. 2021), regardless of the sensory modality in which those changes occur (Mouraux and Iannetti 2009; Liang et al. 2010). In contrast, far less is known about the brain responses elicited by abrupt and unexpected stimulus *offsets*. Consequently, whether *onset* and *offset* VPs reflect the functioning of the same neural system is unknown, limiting our understanding of the functional importance of a large and fundamental phenomenon of the mammalian brain (Bancaud et al. 1953; Knight et al. 1985; Beydoun et al. 1997).

We addressed this problem in four experiments in which we recorded the brain activity from 44 participants while delivering abrupt *onsets* and *offsets*. Crucially, *onsets* and *offsets* were carefully matched with respect to all stimulus features (i.e., abruptness, differential intensity, and unexpectedness) except the direction of the change in intensity. We predicted that if *onsets* and *offsets* elicit VPs reflecting the functioning of the same neural system, then they (1) would have quantitatively highly-similar temporal evolution of their scalp distributions, and (2) would be largely comprised of similar, supramodal components. Additionally, we predicted that, like the *onset* VP, the *offset* VP would (3) be comparably sensitive to temporal unexpectedness, and (4) co-occur with similar activations of the motor system. Overall, we observed a remarkable degree of phenomenological and functional similarity between the brain responses elicited by abrupt *onsets* and *offsets* of both auditory and somatosensory stimuli. This result suggests that these electrocortical responses mostly reflect the activation of a common, supramodal neural network, consequent to the detection of behaviorally-relevant environmental changes.

### Abrupt Onsets and Offsets Activate a Common, Supramodal Brain Network

Experiments 1 and 2 demonstrate that *onsets* and *offsets* of both auditory and somatosensory stimuli elicit highly similar EEG responses in the time domain, dominated by the large negative–positive waves composing the VP. In Experiment 1, we employed a point-by-point spatial correlation to compare the spatial distributions of the *onset* and *offset* responses throughout their timecourse, at much higher spatial and temporal resolution than previous studies (e.g., Yamashiro et al. 2008). We observed that the evolution over time of the response scalp distributions was highly similar across *onset* and *offset*-evoked responses, expanding on a previous study which found similar correlations but restricted their analysis to the response peaks and used a low-density 15-channel EEG system (Yamashiro et al. 2008).

In Experiment 2, we adapted an established method for classifying ERP independent components according to their selectivity for particular conditions (Mouraux and Iannetti 2009; Liang et al. 2010), but improved upon the previously-used binary classification with a less-arbitrary and more quantitative analysis of the selectivity of each independent component (Fig. 4 and 5). This approach demonstrated that the *onset* and *offset* responses elicited in the auditory and somatosensory modalities are largely comprised of similar neural activity, which is supramodal and non-specific to either *onsets* or *offsets*, extending the previous finding to multiple sensory modalities. This clearly does not imply that the neural activity elicited by *onsets* and *offsets* is identical, but given the limited spatial resolution of EEG, the differences between the neural activity underlying *onset* vs *offset* responses are likely to be fine-grained in both the auditory and somatosensory modalities. Indeed, we did find some small independent components which were more selective for one sensory modality (as in previous work: Mouraux and Iannetti, 2009; Liang et al. 2010) or for a particular direction of intensity change. However, not only did these components reflect the smallest proportions of response variance (Fig. 5), but they were also only marginally selective, with no component having a selectivity ratio larger than ~ 3 (see Methods), and were therefore far from being *“specific*” for any particular condition. Thus, these results demonstrate that most of the variance of the auditory and somatosensory *onset*- and *offset*-evoked VPs (i.e., the bulk of the recorded response) was supramodal and non-specific for the direction of the intensity change.

This finding contradicts some common interpretations that *onset* and *offset* responses reflect the detection of intensity changes solely within a particular sensory modality (e.g., Martin and Boothroyd 1999, 2000; Weise et al. 2012, 2018). For example, the VP elicited by changes in auditory intensity has been interpreted by some authors in a modality-specific fashion, and the response consequently labeled as the “auditory change complex” (ACC; Martin and Boothroyd 1999, 2000), an interpretation still pervasive in the clinical literature (Friesen and Tremblay 2006; Hoppe et al. 2010; He et al. 2015; Mathew et al. 2017).

In addition to the phenomenological results of Experiments 1 and 2, the results of Experiments 3 and 4 provide functional evidence that a common network subserves *onset* and *offset* brain responses. Experiment 3 demonstrates that *onsets* and *offsets* are similarly sensitive to the temporal predictability of the eliciting stimulus, with more predictable (and therefore less surprising) stimuli eliciting a smaller brain response (Fig. 7)—a finding consistent with the observation that *offsets* following shortly after the preceding *onset* elicit a smaller-amplitude VP (Davis and Zerlin 1966). The scalp distribution of the response habituation was also similar across *onsets* and *offsets*. This similarity implies that the neural generators sensitive to stimulus surprise were the same in both *onset* and *offset* responses, therefore providing even stronger evidence for a shared neural substrate.

Experiment 4 additionally provides evidence that the VPs elicited by *onsets* and *offsets* co-occur with similar modulations of exerted muscular force: both *onsets* and *offsets* clearly modulated the force output, eliciting a similar increase and subsequent decrease of force. One minor but notable difference was that the force response elicited by the *offset* did not include an initial decrease, as did the *onset* response (Fig. 8). This somehow matches the smaller amplitude of the negative wave of the EEG response elicited by *offsets* in Experiments 2 and 4 (Figs. 3 and 8), although Experiments 1 and 3 resulted in *onset* and *offset* VPs of similar amplitude (Figs. 2 and 7). Thus, while it is difficult to draw definite conclusions, these results altogether suggest that the late positive components of both the EEG and the force are entirely non-specific (i.e., similar in both *onset* and *offset* responses), while the earlier components are, to a certain degree, more often observed in response to *onsets*. Despite the minor difference represented by the lack of early force reduction following *offsets*, both *onset* and *offset* VPs co-occur with clear modulations of muscular activity, suggesting that they both reflect an underlying system closely related to the output of the motor system and pointing towards a similar functional significance of these responses—as discussed in more detail in the following section.

Altogether, these findings suggest that abrupt *onsets* and *offsets* activate a common, supramodal brain network. But what are the neural structures comprising this network? Recently, we argued that VPs reflect the activity of the extralemniscal system (Somervail et al. 2021), an interpretation which was once popular but has since been largely forgotten (e.g. Jasper 1960; Lindsley 1969; Fruhstorfer 1971; reviewed in Näätänen and Picton 1987). Extralemniscal sensory pathways run in parallel to canonical modality-specific lemniscal pathways, and transmit low-fidelity information to supramodal thalamic nuclei that project widely to the cortex and striatum (Hu 2003). Several lines of evidence suggest that the VP is the cortical consequence of the activation of the extralemniscal system. For example, unlike neurons in lemniscal relay nuclei, extralemniscal thalamic neurons respond to stimuli of several modalities (Guilbaud 1968; Albe-Fessard and Besson 1973; Peschanski et al. 1981; Komura et al. 2005), and the VP largely reflects supramodal cortical activity (Mouraux and Iannetti 2009). Both the VP and extralemniscal thalamic responses rapidly habituate to stimuli repeated at short and predictable intervals (Peschanski et al. 1981; Calford and Aitkin 1983; Bordi and LeDoux 1994; Edeline et al. 1999; Iannetti et al. 2008; Anderson et al. 2009). More direct evidence is that general anesthetics abolish extralemniscal responses while leaving lemniscal responses intact, and abolish the VP elicited by auditory stimulation without affecting the modality-specific lateralised EEG responses (Simpson and Knight 1993). In contrast, the VP elicited by sudden auditory stimuli is largely unaffected by bilateral ablation of the primary auditory cortex, while the early lateralised responses were totally abolished (Simpson and Knight 1993). The finding that *onsets* and *offsets* activate a common network provides further evidence that these VPs reflect extralemniscal activity. Indeed, like *onset*- and *offset*-evoked VPs, extralemniscal thalamic neurons respond to sudden and unexpected stimulus *onsets* and *offsets*, but not to sustained or repetitive stimulation (Albe-Fessard and Kruger 1962; Albe-Fessard and Besson 1973; Peschanski et al. 1981).

### Offset-evoked Vertex Potentials do not Merely Encode Changes of Sensory Intensity, but Rather the Behavioral Relevance of those Changes

As mentioned in previous paragraphs, *offset*-evoked VPs have often been interpreted in terms of modality-specific perception. Another naïve interpretation of the VPs elicited by *onsets* and *offsets* is that they merely encode the cortical representation of the beginning and end of a sensory event. However, the results of Experiment 3 demonstrate that the magnitude of the *offset* response does not faithfully represent the intensity drop, but rather its unexpectedness or *surprise* content, which we define here as the degree to which the stimulus violates expectations. This is a function of (1) the particular predictions of the system and (2) the amount by which the stimulus deviates from those predictions. Notably, this is also the case for the more thoroughly investigated *onset* brain response (Iannetti et al. 2008; Wang et al. 2010; Valentini et al. 2011; Ronga et al. 2013).

What is the functional significance of the *offset* responses investigated here? The sensitivity of an ERP to unexpected sensory events can be explained as the encoding of prediction error associated with a violation of expectations (Friston 2005). In this framework, the system underlying the VP may have a number of priors (derived from evolution, experience, or both), such as that no intensity change will occur, and that when an intensity change occurs repeatedly at constant interval, it will continue to occur at the same temporal interval. Thus, monotonously repeated stimuli are more expected and result in a smaller surprise signal (i.e., in a VP of smaller amplitude; Iannetti et al. 2008). The unexpected occurrence of changes in specific stimulus features within the sequence of repeated stimuli (e.g., changes in stimulus intensity, modality, or location) violate this prediction, resulting in another increase of the surprise signal and thereby reversing the VP habituation (i.e., a *dis*habituation; Valentini et al. 2011; Ronga et al. 2013; Moayedi et al. 2016). These priors (or rules) can be studied to determine the system teleology. Indeed, previous studies of the *onset*-evoked VP have revealed that not all types of sensory changes are equally capable of eliciting a surprise signal. For example, the habituation due to the repetition of identical stimuli can be reversed only by changes of particular stimulus properties, such as sensory modality (Valentini et al. 2011), location in egocentric, but not somatotopic, coordinates (Torta et al. 2012; Moayedi et al. 2016) and successive increases, but not decreases, of stimulus intensity in a sequence of abrupt stimuli (Ronga et al. 2013).

The predictions of the system seem to be tuned such that the most surprising sensory changes are those which have more relevance to *urgent* behaviors. For example, the importance of stimuli moving towards the core of the body (Moayedi et al. 2016), or the importance of stimuli becoming sequentially more intense (Ronga et al. 2013) clearly have a higher relevance to survival in a natural environment: they more likely represent a threat to the body which demands immediate attention and behavioral reaction.

Several other lines of evidence link VPs to immediate behavioral reaction: in Experiment 4, both *onsets* and *offsets* were capable of eliciting a specific modulation of muscular activity, possibly to prepare the individual for swift reactions to current or future environmental events (Novembre et al. 2018); furthermore, the VP amplitude has been shown to reliably predict the reaction time of subsequent speeded reactions (Moayedi et al. 2015; Kilintari et al. 2018; Tiemann et al. 2018). Importantly, this relationship is even stronger when the behavior has a more ethological urgency, such as a defensive limb withdrawal rather than an equivalent non-defensive movement (Moayedi et al. 2015). It therefore seems likely that, rather than purely reflecting the sensory-cortical encoding of sudden drops of sensory input, *offset-* (and *onset*-) evoked VPs instead reflect a predictive model which is geared towards the detection of behaviorally-relevant environmental changes, and the preparation for appropriate motoric responses to those changes.
